# Supplementary material for: Community perspectives on maternal dietary diversity in rural Kenya, Mozambique and The Gambia: A PRECISE Network qualitative study
Source: PLOS Glob Public Health. 2025 Apr 2;5(4):e0004411. doi: 10.1371/journal.pgph.0004411 (PMC11964213; doi:10.1371/journal.pgph.0004411)
Supplement: S1 File — (DOCX) [file pgph.0004411.s001.docx]

**S1 File. Semi-structured interview guide**
*(Translated into local languages for each setting)*

**Demographic and opening questions**

1. Do you have any children?

- Boys? Girls? How old are they?

1. Did you grow up in this community?

- Do you have family members in this community?
- For those not born in the community: When did you come here? Why did you move here? (i.e. marriage, economic opportunity, family etc.)

1. What is your ethnic background?
2. How old are you?
3. Did you attend any schooling?

- If so, how many years of school did you complete?

1. What do you do for a living?

- How much do you typically earn in a day?

1. Are you currently married?

- If not: single, unmarried partner, divorced, widowed?
- If married: what does your spouse do for a living? How much do they typically earn in a day? Do they work in the community or have to travel away for work?

1. Can you describe a typical day for you?

**Local diets and food patterns**

1. Please tell me about the typical meals people eat in this community

- What meals do people usually eat everyday in the morning, afternoon and evening?
- Are different foods and meals people eat at different times of the year?
- Are there any foods or meals that women eat especially during pregnancy, delivery, when they are breastfeeding?

*I’m going to go through a list of different food groups and I would like you to tell me what foods are commonly eaten in this community:*

1. Are eggs eaten here?

- Eaten regularly (at least once a week) or not eaten often (less than once a week)?
- Do you eat more eggs in certain months or are they eaten around the year? If in certain months, which months?
- Do pregnant women eat these foods? Why or why not?

1. Is meat and chicken eaten here, including organ meats? What types?

- How frequently for red meats (i.e. Beef, pork, lamb, goat, rabbit, wild game meat), organ meats (i.e. kidney, liver), and poultry (i.e. chicken, duck, any birds)? Eaten regularly (at least once a week) or not eaten often (less than once a week)?
- Do you eat more of these foods in certain months or are they eaten around the year? If in certain months, which months?
- Do pregnant women eat these foods? Why or why not?

1. Is fish and other seafood eaten here? What types?

- Eaten regularly (at least once a week) or not eaten often (less than once a week)?
- Do you eat more of these foods in certain months or are they eaten around the year? If in certain months, which months?
- Do pregnant women eat these foods? Why or why not?

1. Is low fat-fat dairy like milk, cheese, yoghurt or other milk products (NOT butter, cream or sour cream) eaten here? What types?

- Eaten regularly (at least once a week) or not eaten often (less than once a week)?
- Do you eat more of these foods in certain months or are they eaten around the year? If in certain months, which months?
- Do pregnant women eat these foods? Why or why not?

1. Are nuts and seeds like Tree nut, ground nut, peanut or certain seeds or nut/seed "butters" or pastes eaten here? What types?

- Eaten regularly (at least once a week) or not eaten often (less than once a week)?
- Do you eat more of these foods in certain months or are they eaten around the year? If in certain months, which months?
- Do pregnant women eat these foods? Why or why not?

1. Are peas, lentils and beans eaten here? What types?

- Eaten regularly (at least once a week) or not eaten often (less than once a week)?
- Do you eat more of these foods in certain months or are they eaten around the year? If in certain months, which months?
- Do pregnant women eat these foods? Why or why not?

1. Are cruciferous vegetables like cabbage and kale and dark green leafy vegetable eaten here? What types?

- Eaten regularly (at least once a week) or not eaten often (less than once a week)?
- Do you eat more of these foods in certain months or are they eaten around the year? If in certain months, which months?
- Do pregnant women eat these foods? Why or why not?

1. Are white roots and tubers or plantains eaten here? What types?

- Eaten regularly (at least once a week) or not eaten often (less than once a week)?
- Do you eat more of these foods in certain months or are they eaten around the year? If in certain months, which months?
- Do pregnant women eat these foods? Why or why not?

1. Are vegetables or roots that are orange-coloured inside like carrots and sweet potatoes eaten here? What types?

- Eaten regularly (at least once a week) or not eaten often (less than once a week)?
- Do you eat more of these foods in certain months or are they eaten around the year? If in certain months, which months?
- Do pregnant women eat these foods? Why or why not?

1. Any other types of vegetables are eaten here that we have not mentioned?

- Eaten regularly (at least once a week) or not eaten often (less than once a week)?
- Do you eat more of these foods in certain months or are they eaten around the year? If in certain months, which months?
- Do pregnant women eat these foods? Why or why not?

1. Are other fruits that are dark yellow or orange eaten here? What types?

- Eaten regularly (at least once a week) or not eaten often (less than once a week)?
- Do you eat more of these foods in certain months or are they eaten around the year? If in certain months, which months?
- Do pregnant women eat these foods? Why or why not?

1. Any other types of fruits are eaten here that we have not mentioned?

- Eaten regularly (at least once a week) or not eaten often (less than once a week)?
- Do you eat more of these foods in certain months or are they eaten around the year? If in certain months, which months?
- Do pregnant women eat these foods? Why or why not?

1. What kinds of sweets are eaten here, if any?

- Eaten regularly (at least once a week) or not eaten often (less than once a week)?
- When would people eat these foods?
- Do pregnant women eat these foods? Why or why not?

1. Fried snacks and savoury snacks in packets, fast/street foods

- Eaten regularly (at least once a week) or not eaten often (less than once a week)?
- When would people eat these foods?
- Do pregnant women eat these foods? Why or why not?

1. Refined grains and baked goods

- Eaten regularly (at least once a week) or not eaten often (less than once a week)?
- When would people eat these foods?
- Do pregnant women eat these foods? Why or why not?

1. Sugar-sweetened fizzy drinks?

- Eaten regularly (at least once a week) or not eaten often (less than once a week)?
- When would people eat these foods?
- Do pregnant women eat these foods? Why or why not?

1. Any other foods people eat here that we have not mentioned?

- Eaten regularly (at least once a week) or not eaten often (less than once a week)?
- When would people eat these foods?
- Do pregnant women eat these foods? Why or why not?

**Food provisioning and meals**

1. Where do you get your food from? How far do you have to travel to get your food?

- Who goes to get the food items in your family and how far do they have to travel for it? Are there different places for different types of food?
- How do you/people in this community store food?
- How many hours of your day do you (or your family member) spend getting, preparing and cooking food and cleaning after meals?
- Does any of these daily activities change when a woman is pregnant or caring for a young infant?

1. Please share how meals are eaten in your family

- Where does your household eat meals? Does everyone eat together?
- Do certain family members eat first?
- Does everyone in the family eat the same meal? If not, how does it differ and for who?

1. Has food and food related activities changed in your lifetime?

- Do you eat the same food as your parents? Your grandparents? If not, what has changed and why.
- Has anything changed in where you get your food, how you store your food items and how you cook? When has these changes happened and why?

**Local perspectives on food and healthy diets**

1. Are there any foods or meals that are associated to your ethnic group?

- What are your traditional foods that people eat in your community? Do people still eat this? If not, why?
- What are favourite foods or meals that people in this community would eat everyday if they could?
- Are there any foods that people in this community do not eat? Why? (if they simply reply that it’s culture, try to probe deeper)
- Are there any stories or beliefs in this community about foods that pregnant and/or lactating mothers should eat? Why?
- Are there any stories or beliefs in this community about foods that pregnant and/or lactating mothers should not eat? Why?

1. What does a healthy meal mean to you? What makes a meal healthy? Why?

- What is needed to make a meal considered complete? (i.e. if this food item is missing, then the meal is not complete)

1. What foods/meals do you think is healthy for pregnant women to eat? Why? Where did you learn this from?
2. What foods/meals do you think is healthy for a woman who is breastfeeding to eat? Why? Where did you learn this from?

**Barriers and facilitators to adequate maternal diets and health**

1. In your opinion, do you think pregnant and breastfeeding women in this community are able to eat enough food? Eat enough healthy meals? Why or why not?

- What do you think are the key problems that happen when women do not get enough healthy food to eat?
- In your opinion, is there a relationship between the food a woman eats and getting high blood pressure during pregnancy?

1. During the last 12 months, was there a time when, because of lack of money or other resources (probe for examples and stories if possible)

- You were worried you would not have enough food to eat?
- You were unable to eat healthy and nutritious food?
- You ate only a few kinds of foods?
- You had to skip a meal?
- You ate less than you thought you should?
- Your household ran out of food?
- You were hungry but did not eat?
- You went without eating for a whole day?

1. What do women do when there is not enough food in their homes?

- Do mothers change their eating (i.e. eat less) so other people in their families can eat more? Prioritize food for which family members?
- What coping strategies employed?
- Do people gossip and talk bad about women/ households if they know a household cannot get enough food or if a child looks hungry?

1. In your opinion, what helps women have enough food?

- For their household
- For themselves

1. In your opinion, what helps women to have good health during their pregnancy, delivery and caring for their young infant?

**Closing**

1. Is there anything else you would like to tell me that we have not already discussed?

*Thank participants for their contribution.*
