# Supplementary material for: Community perspectives on maternal dietary diversity in rural Kenya, Mozambique and The Gambia: A PRECISE Network qualitative study
Source: PLOS Glob Public Health. 2025 Apr 2;5(4):e0004411. doi: 10.1371/journal.pgph.0004411 (PMC11964213; doi:10.1371/journal.pgph.0004411)
Supplement: S1 Table — (DOCX) [file pgph.0004411.s002.docx]

**S1 Table: The PRECISE Network**

| **In-country teams** | **Members** |
| --- | --- |
| THE GAMBIA: Medical Research Council Unit The Gambia at the London School of Hygiene and Tropical Medicine, Fajara | Umberto D’Alessandro, Anna Roca, Hawanatu Jah, Andrew Prentice, Melisa Martinez-Alvarez, Brahima Diallo, Abdul Sesay, Sambou Suso, Baboucarr Njie, Fatima Touray, Yahaya Idris, Fatoumata Kongira, Modou F.S. Ndure, Lawrence Gibba, Abdoulie Bah and Yorro Bah. |
| KENYA: Aga Khan University, Nairobi | Marleen Temmerman, Angela Koech, Patricia Okiro, Geoffrey Omuse, Grace Mwashigadi, Consolata Juma, Joseph Mutunga, Moses Mukhanya, Onesmus Wanje, Isaac Mwaniki, Marvin Ochieng, Emily Mwadime, Alice Kombo, Nathan Barreh, Christine Dama Baya , Juma Gumbo, Grace Maitha, Irene Chadi and Sharon Konde |
| MOZAMBIQUE : Centro de Investigação em Saúde de Manhiça, Manhiça | Esperança Sevene, Corssino Tchavana, Salesio Macuacua, Anifa Vala, Helena Boene, Lazaro Quimice, Sonia Maculuve, Inacio Mandomando |
| **Central co-ordinating team** |  |
| Department of Women and Children’s Health, School of Life Course Sciences, Faculty of Life Sciences and Medicine, King’s College London | Peter von Dadelszen, Laura A. Magee, Rachel Craik, Marie-Laure Volvert, Hiten Mistry, Thomas Mendy |
| Donna Russell Consulting | Donna Russell |
| **Co-Investigator team** |  |
| Midlands State University, Zimbabwe | Prestige Tatenda Makanga, Liberty Makacha and Reason Mlambo |
| Kings College London | Lucilla Poston, Rachel Tribe, Sophie Moore, Tatiana Salisbury |
| University of Oxford | Aris Papageorghiou, Alison Noble, Rachel Craik |
| London School of Hygiene and Tropical Medicine | Hannah Blencowe, Veronique Filippi, Joy Lawn, Matt Silver, Joseph Akuze and Ursula Gazeley |
| St George’s, University of London | Judith Cartwright, Guy Whitley, Sanjeev Krishna |
| University of British Columbia | Marianne Vidler, Jing (Larry) Li, Jeff Bone, Mai-Lei (Maggie) Woo Kinshella, Domena Tu, Ash Sandhu, Kelly Pickerill |
| Eduardo Mondlane University, Maputo | Carla Carillho |
| Imperial College London | Benjamin Barratt |
