# Supplementary material for: Community perspectives on maternal dietary diversity in rural Kenya, Mozambique and The Gambia: A PRECISE Network qualitative study
Source: PLOS Glob Public Health. 2025 Apr 2;5(4):e0004411. doi: 10.1371/journal.pgph.0004411 (PMC11964213; doi:10.1371/journal.pgph.0004411)
Supplement: S2 Table — (DOCX) [file pgph.0004411.s003.docx]

S1 Table. Qualitative analysis coding framework

| **Codes** | **Sub-codes** | **Description** |
| --- | --- | --- |
| Maternal dietary patterns and dietary diversity | Typical meals | Typical meals for breakfast, lunch, and dinner |
|  | Special diets for pregnant and lactating women | Mentions of foods especially eaten during pregnancy, delivery and breastfeeding |
|  | Consumption patterns | Staple foods, luxury foods, seasonal foods, special occasion foods, snacks and non-nutritive foods for children, other |
|  | Influencing factors | Descriptions of factors that impact the type of foods pregnant women are able to eat |
| Community knowledge of healthy maternal diets | Healthful foods | Foods that are described as healthy for pregnant and lactating mothers |
|  | Unhealthy foods | Foods that are described as unhealthy for pregnant and lactating mothers |
|  | Beliefs about maternal diets | Reasons why for eating or avoiding certain foods |
|  | Associations between maternal diets and health | Local conceptualizations on how maternal diets connect with maternal health, sources of information and knowledge gaps |
| Food security | Experiences of food insecurity | Descriptions of food insecurity, including worry, few foods, unable to get healthy foods, ate less, skipped meals, household ran out of food, hungry but did not eat, did not eat for a whole day |
|  | Experiences of food security | Descriptions of feeling food security in their household |
|  | Coping mechanisms | Methods for coping with food insecurity |
|  | Barriers and facilitators | Factors that impacted availability, accessibility, use/utilization and stability of household food supply |
| Other | Other | Other topics that did not fit existing codes |
