# Supplementary material for: Community perspectives on maternal dietary diversity in rural Kenya, Mozambique and The Gambia: A PRECISE Network qualitative study
Source: PLOS Glob Public Health. 2025 Apr 2;5(4):e0004411. doi: 10.1371/journal.pgph.0004411 (PMC11964213; doi:10.1371/journal.pgph.0004411)
Supplement: S3 Table — (DOCX) [file pgph.0004411.s004.docx]

**S2 Table. Frequency of reported themes by country and overall**

|  | **Number of participants** | | | | **Number of references coded** | | | |
| --- | --- | --- | --- | --- | --- | --- | --- | --- |
|  | **Kenya  (n=18)** | **The Gambia (n=15)** | **Mozambique (n=14)** | **Total (n=47)** | **Kenya** | **The Gambia** | **Mozambique** | **Total** |
| Staple foods | 17 (94%) | 11 (73%) | 13 (93%) | 41 (87%) | 41 | 22 | 35 | 98 |
| Special foods for pregnancy | 18 (100%) | 15 (100%) | 14 (100%) | 47 (100%) | 68 | 90 | 49 | 207 |
| Special foods around delivery | 14 (78%) | 0 (0%) | 8 (57%) | 22 (47%) | 30 | 0 | 15 | 45 |
| Special foods for lactation | 16 (89%) | 12 (80%) | 13 (93%) | 41 (87%) | 32 | 18 | 33 | 83 |
| Value of dietary diversity overall | 16 (83%) | 10 (67%) | 11 (79%) | 37 (79%) | 32 | 15 | 33 | 80 |
| Maternal dietary diversity | 14 (78%) | 10 (67%) | 10 (71%) | 34 (72%) | 40 | 21 | 16 | 77 |
| Feasibility of diverse meals | 11 (61%) | 14 (93%) | 12 (86%) | 37 (79%) | 22 | 33 | 29 | 84 |
| Influencing factors | | | | | | | | |
| Affordability | 18 (100%) | 15 (100%) | 13 (93%) | 46 (98%) | 162 | 77 | 90 | 329 |
| Seasonality | 17 (94%) | 15 (100%) | 13 (93%) | 45 (96%) | 73 | 92 | 83 | 248 |
| Droughts/storms | 12 (67%) | 3 (20%) | 3 (21%) | 18 (38%) | 38 | 3 | 11 | 52 |
| Gender norms | 18 (100%) | 12 (80%) | 10 (71%) | 40 (85%) | 82 | 44 | 23 | 149 |
| Knowledge | 18 (100%) | 15 (100%) | 14 (100%) | 47 (100%) | 61 | 56 | 44 | 161 |
| Cravings | 17 (94%) | 9 (60%) | 14 (100%) | 40 (85%) | 60 | 15 | 68 | 143 |
| Traditional beliefs | 17 (94%) | 10 (67%) | 13 (93%) | 40 (85%) | 58 | 22 | 25 | 105 |
| Religion and celebrations | 16 (89%) | 8 (53%) | 10 (71%) | 34 (72%) | 57 | 12 | 16 | 85 |
| Rural residence | 8 (44%) | 12 (80%) | 4 (29%) | 24 (51%) | 16 | 33 | 12 | 61 |
