# Supplementary material for: Community perspectives on maternal dietary diversity in rural Kenya, Mozambique and The Gambia: A PRECISE Network qualitative study
Source: PLOS Glob Public Health. 2025 Apr 2;5(4):e0004411. doi: 10.1371/journal.pgph.0004411 (PMC11964213; doi:10.1371/journal.pgph.0004411)
Supplement: S4 Table — (DOCX) [file pgph.0004411.s005.docx]

S3 Table. Frequency of themes reported in each participant group, by number of participants

|  | **Kenya** | | | | **The Gambia** | | | | **Mozambique** | | | | **Overall** | | | |
| --- | --- | --- | --- | --- | --- | --- | --- | --- | --- | --- | --- | --- | --- | --- | --- | --- |
|  | PW/ RM (n=7) | MR  (n=4) | FR (n=3) | CL  (n=4) | PW/ RM (n=5) | MR (n=3) | FR (n=3) | CL (n=4) | PW/ RM  (n=5) | MR  (n=3) | FR  (n=3) | CL  (n=3) | PW/ RM  (n=17) | MR  (n=10) | FR  (n=9) | CL  (n=11) |
| Staple foods | 6 | 4 | 3 | 4 | 4 | 2 | 2 | 3 | 4 | 3 | 3 | 3 | 14 | 9 | 8 | 10 |
| Special foods for pregnancy | 7 | 4 | 3 | 4 | 5 | 3 | 3 | 4 | 5 | 3 | 3 | 3 | 17 | 10 | 9 | 11 |
| Special foods around delivery | 4 | 3 | 3 | 4 | 0 | 0 | 0 | 0 | 2 | 1 | 2 | 3 | 6 | 4 | 5 | 7 |
| Special foods for lactation | 5 | 4 | 3 | 4 | 5 | 2 | 3 | 3 | 5 | 2 | 3 | 3 | 15 | 8 | 9 | 10 |
| Value of dietary diversity overall | 5 | 4 | 3 | 4 | 3 | 2 | 2 | 3 | 3 | 2 | 3 | 3 | 11 | 8 | 8 | 10 |
| Maternal dietary diversity | 5 | 3 | 2 | 4 | 5 | 1 | 0 | 4 | 4 | 2 | 3 | 1 | 14 | 6 | 5 | 9 |
| Feasibility of diverse meals | 3 | 3 | 1 | 4 | 4 | 3 | 3 | 5 | 4 | 2 | 3 | 3 | 11 | 8 | 7 | 11 |
| Influencing factors | | | | | | | | | | | | | | | | |
| Affordability | 7 | 4 | 3 | 4 | 5 | 3 | 3 | 4 | 4 | 3 | 3 | 3 | 16 | 10 | 9 | 11 |
| Seasonality | 6 | 4 | 3 | 4 | 5 | 3 | 3 | 4 | 4 | 2 | 3 | 3 | 15 | 9 | 9 | 11 |
| Droughts/storms | 3 | 4 | 1 | 4 | 2 | 0 | 0 | 1 | 1 | 1 | 1 | 0 | 6 | 5 | 2 | 5 |
| Gender norms | 7 | 4 | 3 | 4 | 2 | 3 | 3 | 4 | 2 | 3 | 2 | 3 | 11 | 10 | 8 | 11 |
| Knowledge | 7 | 4 | 3 | 4 | 5 | 3 | 3 | 4 | 5 | 3 | 3 | 3 | 17 | 10 | 9 | 11 |
| Cravings | 6 | 4 | 3 | 4 | 4 | 1 | 2 | 1 | 5 | 3 | 3 | 3 | 15 | 8 | 8 | 8 |
| Traditional beliefs | 6 | 4 | 3 | 4 | 4 | 1 | 1 | 4 | 4 | 3 | 3 | 3 | 14 | 8 | 7 | 11 |
| Religion and celebrations | 7 | 3 | 2 | 4 | 2 | 2 | 1 | 3 | 3 | 1 | 3 | 3 | 12 | 6 | 6 | 10 |
| Rural residence | 2 | 2 | 2 | 2 | 2 | 3 | 3 | 4 | 1 | 1 | 1 | 1 | 5 | 6 | 6 | 7 |

*PW/RM – pregnant woman or recent mother; MR – male relative (husband or brother-in-law); FR – female relative (mother-in-law or sister-in-law); CL – community leader (village elder, village leader, religious leader, traditional birth attendant)*
