# Supplementary material for: Community perspectives on maternal dietary diversity in rural Kenya, Mozambique and The Gambia: A PRECISE Network qualitative study
Source: PLOS Glob Public Health. 2025 Apr 2;5(4):e0004411. doi: 10.1371/journal.pgph.0004411 (PMC11964213; doi:10.1371/journal.pgph.0004411)
Supplement: S5 Table — (DOCX) [file pgph.0004411.s006.docx]

S4 Table. Community perspectives on influencing factors by country

|  | **Kenya** | **The Gambia** | **Mozambique** |
| --- | --- | --- | --- |
| Affordability | “I did not have money [in my last pregnancy] and the food I had was only one type. I would always eat ugali (stiff maize porridge) and omena (small dried fish)” *Recent mother* | “There are specific types of food that pregnant women and lactating others should depend on but due to lack of affordability, we all depend entirely on rice and coos (millet)…” *Spouse* | “She doesn't have because it can be those such things, those vegetables happen that one has to buy while one has no money.” *Recent mother* |
| Seasonality | “During the rainy season, we have many green vegetables and eat a lot of greens…There are also guavas and other trees that produce fruits. You can just grab them while passing by and eat.” *Recent mother* | “At this moment, it is not easy [to get] food as it is rainy season. You can see some have coos (millet), some groundnuts but others don’t have anything. For some they must buy before they can eat, which is not easy.” *Recent mother* | “In summer, we don't have vegetables… The months of April, May and June, those are the winter period….Until July, we still have something. In August, there is nothing in the farms” *Mother-in-law* |
| Irregular weather | “The problem is like now when there is drought. There is no rain at all there is no food in the shambas (farms) so you can not get healthy foods.” *Spouse* | “Today my biggest constraint is this house because last year’s storm destroyed part of it” *Community leader* | “Because there is no rain and there is drought, so much so that now we [just] eat beans” *Mother-in-law* |
| Gender norms | “For fish, they have to be brought for her (pregnant woman) to eat…When it is not brought, it's just like that. Poor her if it is not brought. She will completely not get (any fish).” *Community leader* | “If available, they (women) eat and if not, they just sit and wait… That is it… It is your husband who feeds and protects you.” *Traditional birth attendant* | “There's no money to buy those foods… I only have money at the end of the month… when my husband gives it for the household…. It's not always that he gives me money to buy curry (stew)” *Recent mother* |
| Knowledge | “Whenever you go to the hospital, the doctor advices to eat fruits, vegetables and soup this helps a lot with increasing blood and energy” *Recent mother* | “As a woman, I am talking from experience. I have also gone through the same experience before. I know the type of foods I use to eat during pregnancy.” *Mother-in-law* | “I didn't learn it anywhere. I just have knowledge, and I know that those foods are good for pregnant women and I, in particular, ate those foods when I was pregnant” *Community leader* |
| Cravings | “For pregnant women, you cannot choose for them food. They are sometimes picky and would only eat what they crave for.” *Spouse* | “My mind used to urge me to eat sweets” *Pregnant woman* | “I have a daughter who during her pregnancy she didn't eat anything other than oranges…That's because it's not the woman's will…But…what she carries in her womb.” *Community leader* |
| Traditional beliefs | “A pregnant woman should not eat eggs because they will give birth to a big baby.” *Community leader* | “Many pregnant women are afraid to eat much bread … It makes the baby too big in the womb, yes” *Brother-in-law* | “What they said in the old days was myth… she can eat eggs…because it provides vitamins for the baby.” *Mother-in-law* |
| Religion and celebrations | “We eat meat during Eid because my husband’s family is Muslim and they celebrate Eid. For me because I am Christian I can cook chicken for Christmas.” *Pregnant woman* | “Well [it] is only during feasts such as Tobaski that people usually have enough meat to eat.” *Brother-in-law* | “Pork and cow are things that are consumed a lot when the end of the year comes or on holidays” *Spouse* |
| Rural/urban residence | “It is not easy to find…unless you come to Mariakani (town)” *Mother-in-law* | “We do not have oranges here. If you go to the urban areas and you see it there, you [can] buy… Pregnant women, when they go for the antenatal clinics and see it being sold, they buy and eat.” *Community leader* | “Fruits are consumed but… the trucks park in the village [sell]… a bowl at 50 meticais [and] when someone buys to resells here, an orange is 10 meticais. So for you to buy an orange is difficult.” *Spouse* |

**`**
