# Supplementary material for: Community perspectives on maternal dietary diversity in rural Kenya, Mozambique and The Gambia: A PRECISE Network qualitative study
Source: PLOS Glob Public Health. 2025 Apr 2;5(4):e0004411. doi: 10.1371/journal.pgph.0004411 (PMC11964213; doi:10.1371/journal.pgph.0004411)
Supplement: Attachment — Submitted filename: Response letter_26Jan2025.pdf [file pgph.0004411.s010.pdf]

THE UNIVERSITY OF BRITISH COLUMBIA

Faculty of Medicine
The University of British Columbia
317-2194 Health Sciences Mall
Vancouver, BC V6T 1Z3

Phone 604 822 2421
Fax 604 822 6061
www.med.ubc.ca

Re: GPH-D-24-01813 Community perspectives on maternal dietary diversity in rural Kenya, Mozambique and The Gambia: a PRECISE Network qualitative study
PLOS Global Public Health

Thank you for the positive comments and we are glad that the editor and reviewer found the manuscript “well-written”, the methods “robust”, and the analysis “well conducted”. We appreciate the constructive comments, which were well-taken strengthened the manuscript. We have added an additional paragraph and table in the results to report photographic data from photovoice, to reinforce perceptions of maternal dietary diversity findings from the interviews. Additionally, we have reworked the background and conclusion sections to better highlight public health implications of the findings. Details of how we addressed each comment are described below.

NOTE: The reviewers’ comments are in black font and our responses are in blue font.

COMMENTS

Journal Requirements:

1. We do not publish any copyright or trademark symbols that usually accompany proprietary names, eg (R), (C), or TM (e.g. next to drug or reagent names). Please remove all instances of trademark/copyright symbols throughout the text, including ® on page 26.
    - ® has been removed from the reference.

Additional Editor Comments (if provided):

This paper examines maternal dietary diversity in three countries: Kenya, The Gambia and Mozambique. The authors found that many community members understood the importance of dietary diversity for pregnant women but highlighted affordability and food insecurity due to climate change as reasons why pregnant women are not always able to eat healthy meals. Overall, the paper is well written and the research methods used appropriate. However, I would like to echo, and add to the reviewer's comments.

1. Study significance/"so what": the authors need to more strongly articulate the importance of the study and of their study findings. This can be done by revising the study goals to make them more compelling. Currently, the study goals seem to be aimed at presenting community perspectives on maternal dietary diversity but there is not much explanation of why. The introduction could do a better job at telling the reader what the maternal malnutrition or maternal food insecurity is globally and in the context of the three countries. Is data on this available? Furthermore, the authors should say why this topic is important: is lack of dietary diversity linked to

adverse child health outcomes (e.g., low birth weight) or to poor maternal health outcomes (delivery complications etc)? Please expand your background section so it provides this critical context.

- We added that, "Substantial gaps in meeting minimum dietary diversity for women have been reported in sub-Saharan Africa, including 31% of women with inadequate dietary diversity in Kenya and 48% in Mozambique between 2021-2022 (13)". No data currently exists for The Gambia. This will give more context to the following lines on the adverse pregnancy outcomes associated with a lack of dietary diversity, "Low maternal dietary diversity during pregnancy has been associated with a number of adverse pregnancy outcomes such as increased risk of maternal anemia, preterm birth, and delivering small-for-gestational age or low birthweight infants (14–18), as well as with increased rates of maternal depression and anxiety (19). See lines 61-67.
- Additionally, we have reworked the conclusion to highlight public health implication of the findings: "Our research in Kenya, Mozambique and The Gambia found that dietary diversity was consistently considered by community members important for healthy maternal diet, which supports nutrition education and counselling during pregnancy as recommended by the WHO (20). However, meals for pregnant and recent mothers were usually similar to their households and consisted of a limited number of foods. Moreover, there may be potential misconceptions around dietary diversity and the consumption of low nutrient density foods. Appropriate nutrition knowledge and accessibility are both needed to ensure women have adequate diverse maternal diets to support maternal and perinatal health" (lines 430-437).

2. As per the reviewer's comments, can you present some of the photo voice data and some of the ethnographic data? This may help the reader gain a better sense of the actual diets consumed by pregnant women in the communities concerned.
    - Thank you for the opportunity to elaborate. We have added a new paragraph and an additional "Table 2. Examples of maternal diets described by women during photovoice". Table 2 highlights six photographs of food and meals, with accompanying descriptive quotes, which we agree will better illustrate actual diets and meals consumed by pregnant women in study communities. The aim of the additional qualitative methods was to support credibility of interview findings, as described in the methods (lines 178-181). This is revisited in Table 2 and the new paragraph in the results, "Interview results on perceptions of healthy diverse maternal diets were reinforced by similar findings from photovoice, as illustrated in Table 2. Through the photographs

women took, they described the value of balanced meals with multiple food groups in Kenya, varying meals in Mozambique, and health benefits of dishes with more ingredients in The Gambia" (lines 258-261).

- with six photographs of food and meals from photovoice as well as

3. I also invite you to consider the specific feedback provided by the reviewer.
   - We have addressed each comment, as further detailed below.

[Note: HTML markup is below. Please do not edit.]

Reviewer's comments

Reviewer's Responses to Questions
Comments to the Author

1. Does this manuscript meet PLOS Global Public Health's publication criteria? Is the manuscript technically sound, and do the data support the conclusions? The manuscript must describe methodologically and ethically rigorous research with conclusions that are appropriately drawn based on the data presented.

Reviewer #1: Yes

---

2. Has the statistical analysis been performed appropriately and rigorously?

Reviewer #1: Yes

---

3. Have the authors made all data underlying the findings in their manuscript fully available (please refer to the Data Availability Statement at the start of the manuscript PDF file)?

The PLOS Data policy requires authors to make all data underlying the findings described in their manuscript fully available without restriction, with rare exception. The data should be provided as part of the manuscript or its supporting information, or deposited to a public repository. For example, in addition to summary statistics, the data points behind means, medians and variance measures should be available. If there are restrictions on publicly sharing data—e.g. participant privacy or use of data from a third party—those must be specified.

Reviewer #1: No

4. Is the manuscript presented in an intelligible fashion and written in standard English?

PLOS Global Public Health does not copyedit accepted manuscripts, so the language in submitted articles must be clear, correct, and unambiguous. Any typographical or grammatical errors should be corrected at revision, so please note any specific errors here.

Reviewer #1: Yes

5. Review Comments to the Author

Please use the space provided to explain your answers to the questions above. You may also include additional comments for the author, including concerns about dual publication, research ethics, or publication ethics. (Please upload your review as an attachment if it exceeds 20,000 characters)

Reviewer #1:

1. The research methods are robust and analysis is well conducted. Results of the Ethnographic research part of the study has has not been sufficiently described. Could photographic data collected in study area be presented to the readers?. Especially the data on usual types of food consumed and raw materials sold in market. Do markets sell mostly carbohydrate related food?
    - Thank you for your review and we are pleased to hear that you found the research methods robust and analysis well conducted. We have added additional photographic data in a new Table 2 with six photographs of food and meals taken by women during photovoice, accompanied by their descriptions of the photographs. This will better illustrate the usual types of food and meals consumed by pregnant women in study communities. The photographs mention that fish is bought while some vegetables are grown, though a full description of the foods cultivated and sold in local markets is beyond the scope of the current manuscript and will be further explored in a future paper.
2. Pricing details of foods can be used to triangulate the findings in the affordability theme. Price inflation and Climate change impact can be mentioned in discussion. How does the community perceive affordability with regards to carbohydrates and other nutrients- do they feel hunger is to be satisfied first and then we can worry about the nutrients? More specifically as women- who are the usual cooks at home, what additional

tasks, the women have to do to make the diet diverse, inside the kitchen. if this data is not available, authors can describe in study settings.

- Great point. While our study did not systematically collect pricing details of food, there is a paragraph in the discussion that elaborated on these points (see lines 371-382). To make the point more explicitly, we have revised the following line, "Carbohydrate staples of lower nutritive value were prioritized as fundamental components of meals to feel full, in comparison to costly nutritious foods like fruit, eggs, meat and out-of-season vegetables."

3. Socio-political milieu of the study settings in relation to dietary diversity such as Government schemes, Ethnicity and social hierarchies, civil conflicts, NGOs and projects related maternal nutrition needs mention
   - Ethnicity was associated with local language groups in study communities, which were described as Mijikenda in Kenya, Mandinka, Fula, and Wolof in The Gambia, and Changana in Mozambique in the methods (line 131-133). Swahili and Portuguese are the national languages in Kenya and Mozambique, respectively. Participants did not discuss much about the specifics about social hierarchies, government schemes, civil conflicts, NGOs and other programs in relation to dietary diversity and maternal nutrition, though future research could examine these topics explicitly.
4. Was there any observed difference between primi and multigravida with regards to the study objectives?
   - This is a very interesting question. Our study did not find any observed differences between women who were primigravida or multigravida, but our study design did not specifically seek to understand this research question.
5. Authors mention gender norms. However there is no description of gender norms around cooking, household tasks related to cooking, meal planning and dish washing associated with study objectives. Pregnancy craving is mentioned but was nausea mentioned as a barrier to eating healthy. Also is there a practice of men eating before women even during pregnancy? do children eat before women? are the best served for husband and children? Was this practice described by the participants? if this data is not available, authors can describe in study settings.
   - Thank you for highlighting this important point. In this paper, participants highlighted the gender norms around meal provisioning, particularly how men were seen as the primary providers and decision-makers for the funds to purchase foods (lines 318-320). This

meant that while women may receive maternal nutrition education during antenatal care, there is need for further research and policies to engage men in maternal nutrition education and the economic empowerment women (lines 407-409). We have added the following recommendation for future research in the discussion, "In addition to gender norms around food provisioning, further exploration on the intersections between maternal diets and gender norms around food preparation, household responsibilities, and meal consumption are recommended" (lines 409-411).

6. What is the public health implication of the conclusion of the study?
    - To the best of our understanding, this study is the first to specifically explore local conceptualizations of dietary diversity within the context of maternal diets. We have reworked the conclusion to highlight public health implication of the findings: "Our research in Kenya, Mozambique and The Gambia found that dietary diversity was consistently considered by community members important for healthy maternal diet, which supports nutrition education and counselling during pregnancy as recommended by the WHO (20). However, meals for pregnant and recent mothers were usually similar to their households and consisted of a limited number of foods. Moreover, there may be potential misconceptions around dietary diversity and the consumption of low nutrient density foods. Appropriate nutrition knowledge and accessibility are both needed to ensure women have adequate diverse maternal diets to support maternal and perinatal health" (lines 430-437).

Thank you and we look forward to hearing from the PLOS Global Public Health editorial team.

Best regards,

Mai-Lei Woo Kinshella | Department of Obstetrics and Gynaecology, BC Children's and Women's Hospital and University of British Columbia, Clinical Services Building, Room V3-344, 950 West 28th Ave Vancouver British Columbia, Canada, V5Z 4H4 | Email: Maggie.kinshella@cw.bc.ca
